# Associations of types of grains and lifestyle with all-cause mortality among Chinese adults aged 65 years or older: a prospective cohort study

**DOI:** 10.1186/s12967-023-03927-9

**Published:** 2023-02-06

**Authors:** Yongjie Chen, Boran Sun, Caihong Wang, Keming Zhang, Wenli Lu, Guowei Huang

**Affiliations:** 1 Department of Epidemiology and Statistics, School of Public Health, Tianjin Medical University, No 22 Qixiangtai Road, Tianjin, 300070 China; 2 Department of Nutrition, First Affiliated Hospital of Tianjin University of Traditional Chinese Medicine, Tianjin, China; 3 Department of Nutrition & Food Science, School of Public Health, Tianjin Medical University, No 22 Qixiangtai Road, Tianjin, 300070 China; 4 Tianjin Key Laboratory of Environment, Nutrition and Public Health, Tianjin, China

**Keywords:** Total grains, Staple food, Lifestyle profiles, All-cause mortality, Older adults

## Abstract

**Background:**

Little is known on the association of types of grains with mortality and the moderating effect of lifestyle on this association. This study aims to evaluate the single or joint associations of types of grains and lifestyle with all-cause mortality among Chinese older adults.

**Methods:**

Data were derived from the Chinese Longitudinal Healthy Longevity Survey (CLHLS) from 1998 to 2018. Subjects aged ≥ 65 years were eligible. The types of grains included wheat, total rice, and coarse cereals. Lifestyle was derived using smoking, alcohol consumption, physical activity, and dietary pattern. All-cause mortality was the primary outcome.

**Results:**

This study included 30275 participants with a mean age 87 ± 11 years and documented 19261 deaths during a mean follow-up of 4.8 years. Compared to wheat, in those with healthy and intermediate lifestyle, total rice was associated with a 13% (*HR*: 0.87, *95% CI* 0.80, 0.93) and 6% (*HR*: 0.94, *95% CI* 0.90, 1.00) lower risk of mortality, respectively, and coarse cereals were associated with a 14% (*HR*: 0.86, *95% CI* 0.74, 1.00) and 12% (*HR*: 0.88, *95% CI* 0.79, 0.97) lower risk of mortality, respectively. Meanwhile, an increase per SD in intakes of wheat and coarse cereals was associated with a 10% (*HR*: 1.10, *95% CI* 1.03, 1.18) and 25% (*HR*: 1.25, *95% CI* 1.08, 1.44) higher mortality rate in those with healthy lifestyle, and a 13% (*HR*: 1.13, *95% CI* 1.08, 1.19) and 29% (*HR*: 1.29, *95% CI* 1.17, 1.44) higher mortality in females but not males. In addition, a U-shaped association of intake of total grains with all- cause mortality was observed (*P* for non-linearity = 0.002), and a J-shaped association of intake of total rice with all- cause mortality was observed (*P* for non-linearity = 0.003).

**Conclusions:**

Specific types of grains and lifestyle were separately or jointly associated with all-cause mortality. Compared to wheat, total rice and coarse cereals were advanced grains for participants with a relatively healthy lifestyle. Intake of total rice was related to all-cause mortality in a dose–response manner. Therefore, a combination of intermediate intake of total rice and healthy lifestyle should be encouraged in older adults.

**Supplementary Information:**

The online version contains supplementary material available at 10.1186/s12967-023-03927-9.

## Background

Grains are considered as one of staple foods worldwide [1]. Given the important role of grains in most diets, more and more attentions are paid to the health effect of grains consumption [2]. Many epidemiologic studies and meta- analyses have been conducted to evaluate the relationships between grains consumption and health outcomes, such as cardiovascular disease (CVD), diabetes, cancer, mortality, and longevity [3–8]. However, the purposes of most studies were to assess the associations of whole grains consumption with health outcomes. Few evidences are available on the impacts of specific types of grains on health outcomes. In China, rice and wheat are the major components of grains consumption. Assessing the relationships between specific types of grains and mortality can contribute to guiding consumers to optimize dietary to reduce the risk of mortality.

On the other hand, lifestyle factors account for the majority of many chronic diseases and mortality [9–11]. It is estimated that unhealthy lifestyle factors, such as smoking, heavy alcohol consumption, physical inactivity, and poor diet, contribute to at least 60% of deaths, which in turn lead to a loss of 7.4–17.9 years in life expectancy [12, 13]. Furthermore, previous study reported that lifestyle characteristics modified the association of grains consumption with mortality [5]. Therefore, it is speculated that there is interaction between grains consumption and lifestyle factors on mortality. Given the existing studies on the associations of grains intake and lifestyle factors with mortality, there are still some important gaps. First, few evidences are available on the association of types of grains with mortality. Second, few studies have assessed dietary intake as an aggregative indicator, which is used to define a comprehensive lifestyle profile. Third, little is known on the interactional or joint associations of types of grains and lifestyle with mortality.

To fill the knowledge gaps mentioned above, this study used data from the Chinese Longitudinal Healthy Longevity Survey (CLHLS) to examine the associations of types of grains and a combined lifestyle profile with mortality and evaluate the extent of interactional or joint associations of types of grains and lifestyle profile with all-cause mortality among Chinese older adults. Therefore, the purpose of this study is to evaluate the single and joint associations of types of grains and lifestyle with all-cause mortality and the dose–response associations of intakes of grains with all-cause mortality in older adults. An intensive understanding of how the association of types of grains with mortality varies with lifestyle profile is of benefit to public health.

## Methods

### Study design and sample

Data of this study were derived from the CLHLS, which is an ongoing nationwide longitudinal study. The first survey of the CLHLS was launched in 1998 with follow-up surveys every 2 to 3 years. The latest survey was updated to wave 2018. A multistage, stratified cluster sampling method was used to recruit community dwelling older adults from the sampled villages or communities, which were randomly selected within 631 cities and counties in 22 out of 31 provinces/municipalities in China.

The CLHLS oversampled the oldest old and included 19.5 thousands centenarians, 26.8 thousands nonagenarians, and 29.7 thousands octogenarians. Due to the high mortality rate, new participants were recruited to replace the deceased and lost-to-follow-up respondents in each follow-up wave following the same sex and similar age. Loss to follow-up from each survey year to the next ranged from 8.3% to 20.6% [14]. More details of the study design and the sampling procedure have been published elsewhere [15, 16].

**Fig. 1:**
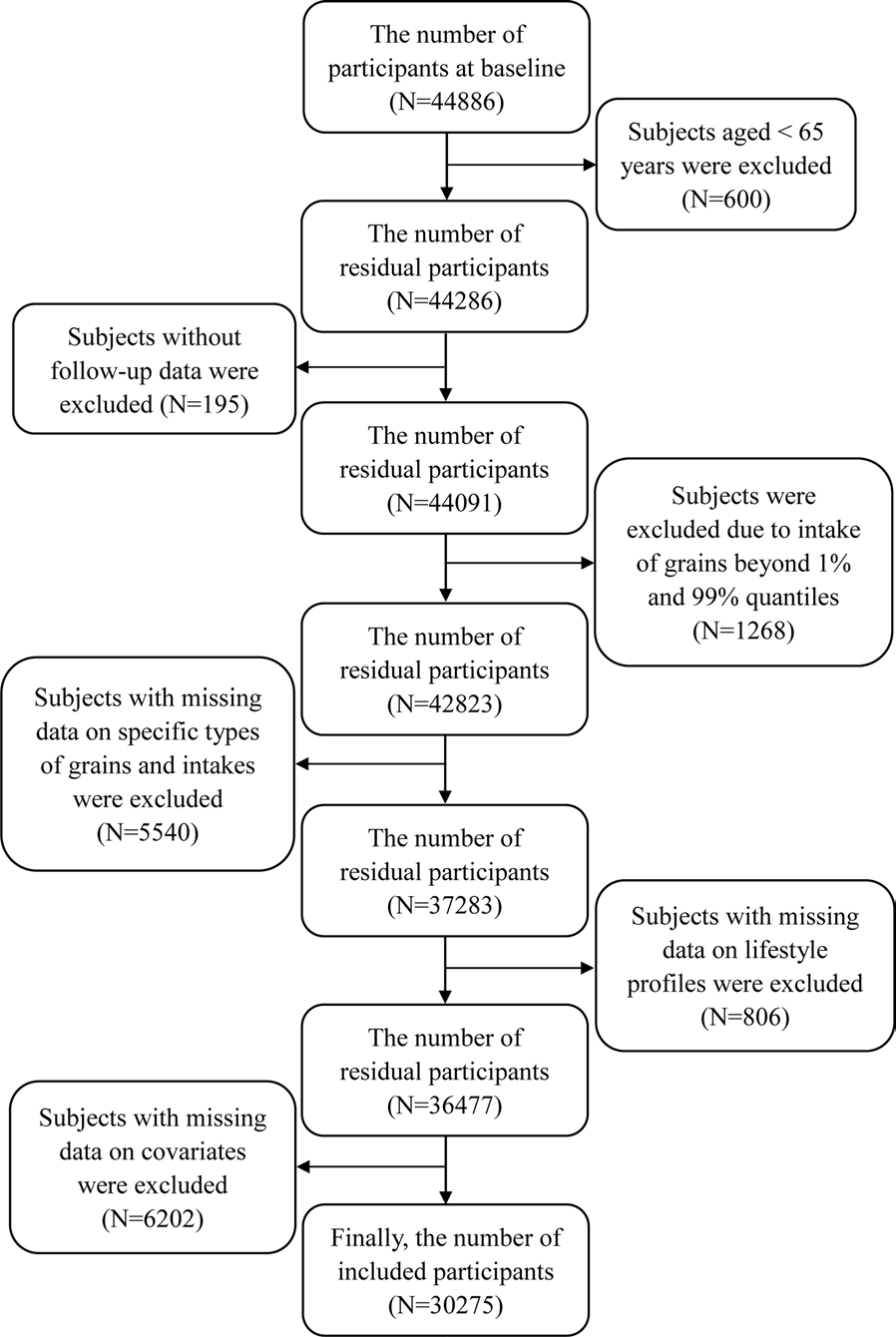
The flow chart of this study

The eligible subjects in this study should meet the following inclusion criteria: subjects were 65 years or older at baseline; and subjects had complete data. The detailed exclusion criteria were as follows: subjects were excluded due to age < 65 years (n = 600); subjects were excluded due to lack of follow-up data (n = 195); subjects were excluded due to grains intake beyond 1% and 99% of quantiles (n = 1268); and subjects were excluded due to missing data of types and intakes of grains (n = 5540), lifestyle profile (n = 806), and covariates (n = 6202). Since there was no follow-up from the 2018 wave yet, participants who firstly enrolled at the 2018 wave were not included in the present study. Finally, a total of 30275 participants were included in this study as shown in Fig. 1.

This study was approved by the Institutional Review Board, Duke University (Pro00062871), and the Biomedical Ethics Committee, Peking University (IRB00001052-13074). All participants provided written informed consent.

### Assessment of types and intakes of grains

The types of grains were assessed using a question of “What is main grain as staple food at present” and were categorized as rice, coarse cereals, and wheat. Since no further questions were used to distinguish between white rice and brown rice and between refined grains and whole grains, the types of grains were named as total rice, coarse cereals, and wheat in this study. Total rice included white rice and rice products (eg, rice cake and rice noodles). Coarse cereals included maize, millet, buckwheat, sorghum, barley, adlay, oat, purple rice, and their associated products. Wheat included wheat flour and its products (eg, steamed bread, noodles, and pancakes). Another question of “How much grains do you eat on average every day?” was used to identify the intake of grains.

### Assessment of healthy lifestyle

A comprehensive lifestyle profile was determined using smoking, alcohol consumption, physical activity, and dietary pattern. According to previous study, body mass index (BMI) could be an intermediate factor between other behavioral factors and mortality [17]. Furthermore, there is an obesity paradox that overweight and obesity might not be strongly associated with mortality in adults [18–21]. Therefore, BMI was not used to define lifestyle profile in this study. Similar to previous study, [22] smoking status was defined as current smokers, former smokers, and never smokers using 2 related questions. Alcohol consumption status was defined as binge drinkers, moderate drinkers, and never drinkers using 3 related questions. Since there were few moderate drinkers (n = 4), moderate drinkers were combined with binge drinkers. Finally, alcohol consumption status was categorized as drinking or no drinking. Physical activity was defined as current, former, and never physical activity using 2 related questions. The detailed assessment of lifestyle factors is provided in Additional file 1: Table S1. According to the relationships between specific types of diet and all-cause mortality in this study (Additional file 1: Table S2), fruits, vegetables, fish, eggs, bean products, and tea were used to define dietary pattern. Since the association of bean products with all-cause mortality was close to significant (*P* = 0.053 and 0.09), bean products were included in the analysis. All types of diet were categorized as “always or almost every day,” “sometimes or occasionally,” or “rarely or never” using a 3-point scale question. The latent class analysis was used to create dietary pattern using PROC LCA in SAS 9.4 [23]. According to the item-response probabilities, dietary pattern was determined as three latent classes characterized by an unfavorable, intermediate, or favorable diet. Then, smoking, alcohol consumption, physical activity, and dietary pattern were used to identify lifestyle profile as healthy, intermediate, or unhealthy lifestyle using latent class analysis. Additional file 1: Supplementary method provides the detailed description of latent class analysis for dietary pattern and lifestyle profile.

### Outcome ascertainment

In each follow-up wave, trained interviewers verified the vital status of all participants from officially issued death certificates whenever available. Otherwise, the vital status was confirmed by a close family member or village doctor. For deceased participants, date of death was further ascertained. All surviving participants were re-interviewed in the next follow-up wave. Duration of follow-up was calculated as the number of years from baseline to death or the end of follow-up, whichever occurred first. Participants surviving until the last survey wave were considered as censoring [24].

### Covariates

In this study, the potential confounders included age, sex, body weight, ethnicity (Han vs others), living areas (rural vs urban), education level (illiteracy vs primary school or above), marital status (married, divorced, widowed, and unmarried), self-reported health (poor, fair, and good), history of hypertension, diabetes, CVD, and coronary heart disease (yes vs no). All information were collected by trained interviewers through face-to-face.

### Statistical analysis

Normality of continuous variables was tested using Kolmogorov–Smirnov. Baseline characteristics are described as mean ± standard deviation (SD) if normal distribution, frequency (percentages) if categorical data. *Analysis of variance* and *chi-square* test were used to compare the differences across specific types of grains in all baseline characteristics for continuous and categorical variables, respectively. Cox proportional hazard regression models were employed to estimate the hazard ratios (*HRs*) and 95% confidence intervals (*CIs*), which were used to assess the associations of types (wheat as the reference) and intakes (increase per SD) of grains and lifestyle profile (healthy lifestyle as the reference) with all-cause mortality. The proportional hazards assumption was tested using Schoenfeld residuals for each exposure and covariate. All models adjusted for age, sex, body weight, ethnicity, living areas, education levels, marital status, history of hypertension, diabetes, CVD, and coronary heart disease, and self-reported health. Meanwhile, Cox frailty models were built with a random term to account for clustering by waves. Furthermore, the sampling weight of the CLHLS was taken into account to correct non-responses bias and over-sampling of certain populations. Further stratified analyses by age (65–79 years vs 80 years or older) and sex (males vs females) were conducted.

Latent class analysis was employed to identify dietary pattern and lifestyle profile. To quantify the multiplicative and additive interactions between types of grains and lifestyle profile, a product term of types of grains and lifestyle profile was additionally added in the model. The relative excess risk due to interaction (RERI) and corresponding *95% CIs* were used to measure the additive interaction, which was described in detail in literature [25]. To evaluate the joint associations of types of grains and lifestyle profile with all-cause mortality, participants were divided into nine groups according to types of grains (wheat, total rice, and coarse cereals) and lifestyle profile (healthy, intermediate, and unhealthy). Hazard ratios of all-cause mortality in different groups were estimated compared to those with wheat as staple food and healthy lifestyle. Finally, restricted cubic splines with three knots (at the 5th, 50th, and 95th percentiles) were used to assess the potential non-linear association of intakes of grains with all- cause mortality.

To test the robustness of the results, three sensitivity analyses were conducted. First, since lifestyle profile could be influenced by major chronic diseases, participants with prevalent hypertension, diabetes, CVD, coronary heart disease, cancer, chronic bronchitis, emphysema, or chronic obstructive pulmonary disease were excluded [26]. Second, participants died within 2 year after baseline were excluded to reduce potential reverse causation. Third, multiple imputation with chained equations was used to impute all missing data of exposures and covariates to test the influence of missing data [27].

All analyses were conducted using SAS 9.4 (SAS Institute Inc., Cary, NC, USA.). A two-tailed *P* ≤ 0.05 indicated a statistical significance.

## Results

### Characteristics of participants at baseline

As Table 1 shown, among 30275 participants (mean age 87 years [SD: 11 years], 43% male), 23486 (78%) were 80 years or older, 22570 (75%) had total rice as staple food, 1317 (4.4%) had coarse cereals as staple food, and 6388 (21%) had wheat as staple food. There were 3622 (12%) participants with unhealthy lifestyle, 18268 (60%) with intermediate lifestyle, and 8385 (28%) with healthy lifestyle. 19261 deaths were recorded during a mean follow-up of 4.8 years. Significant differences across specific types of grains were observed in all baseline characteristics, except sex (*P* = 0.052), alcohol consumer status (*P* = 0.78), and physical activity (*P* = 0.13). Furthermore, unhealthy lifestyle was more prevalent among adults with wheat as staple food. The baseline characteristics of participants excluded due to missing data are shown in Additional file 1: Table S3.

**Table 1:** Baseline characteristics of all participants

Characteristics	Total samples (N = 30275)	Types of grains			
		Total rice (N = 22570)	Coarse cereals (N = 1317)	Wheat (N = 6388)	*P*
Age (years, mean ± SD)^a^	87 ± 11	87 ± 11	88 ± 11	87 ± 11	0.001
Body weight (kg, mean ± SD)^a^	49 ± 11	48 ± 11	50 ± 12	52 ± 12	< 0.001
Intakes of grains (g/d, mean ± SD)^a^	300 ± 130	290 ± 120	340 ± 140	340 ± 140	< 0.001
Sex (n (%))^b^					0.052
Male	13029(43)	9681(43)	535(41)	2813(44)	
Female	17246(57)	12889(57)	782(59)	3575(56)	
Marital status (n (%))^b^					< 0.001
Married	9158(30)	6706(30)	374(28)	2078(33)	
Divorced	153(0.5)	130(0.6)	3(0.2)	20(0.3)	
Widowed	20632(68)	15476(69)	928(70)	4228(66)	
Unmarried	332(1.1)	258(1.1)	12(0.9)	62(1.0)	
Living areas (n (%))^b^					< 0.001
Urban	12173(40)	9618(43)	531(40)	2024(32)	
Rural	18102(60)	12952(57)	786(60)	4364(68)	
Education (n (%))^b^					< 0.001
Illiteracy	19138(63)	13832(61)	898(68)	4408(69)	
Primary school or above	11137(37)	8738(39)	419(32)	1980(31)	
Ethnicity (n (%))^b^					< 0.001
Han	28283(93.4)	20959(92.9)	1149(87.2)	6175(96.7)	
Others	1992(6.6)	1611(7.1)	168(12.8)	213(3.3)	
Smoking status (n (%))^b^					< 0.001
Current	5747(19)	4222(19)	232(18)	1293(20)	
Former	4240(14)	3069(14)	153(12)	1018(16)	
Never	20288(67)	15279(67)	932(70)	4077(64)	
Alcohol consumer status (n (%))^b^					0.78
Yes	6280(21)	4700(21)	265(20)	1315(21)	
No	23995(79)	17870(79)	1052(80)	5073(79)	
Physical activity (n (%))^b^					0.13
Current	8765(29)	6533(29)	400(30)	1832(29)	
Former	1993(6.6)	1529(6.8)	81(6.2)	383(6.0)	
Never	19517(64)	14508(64)	836(63)	4173(65)	
Dietary pattern (n (%))^b^					< 0.001
Unfavorable	7426(25)	5471(24)	339(26)	1616(25)	
Intermediate	13506(45)	10678(47)	553(42)	2275(36)	
Favorable	9343(30)	6421(29)	425(32)	2497(39)	
Lifestyle profiles (n (%))^b^					< 0.001
Unhealthy	3622(12)	2598(12)	142(11)	882(14)	
Intermediate	18268(60)	13691(61)	787(60)	3790(59)	
Healthy	8385(28)	6281(27)	388(29)	1716(27)	
Self-reported health (n (%))^b^					< 0.001
Poor	3857(13)	2879(13)	145(11)	833(13)	
Fair	10230(34)	8007(35)	442(34)	1781(28)	
Good	16188(53)	11684(52)	730(55)	3774(59)	
History of hypertension (n (%))^b^					< 0.001
No	12665(42)	9743(43)	567(43)	2355(37)	
Yes	17610(58)	12827(57)	750(57)	4033(63)	
History of diabetes (n (%))^b^					0.002
No	29721(98.2)	22174(98.3)	1276(96.9)	6271(98.2)	
Yes	554(1.8)	396(1.7)	41(3.1)	117(1.8)	
History of CVD (n (%))^b^					< 0.001
No	29106(96.1)	21813(96.7)	1260(95.7)	6033(94.4)	
Yes	1169(3.9)	757(3.3)	57(4.3)	355(5.6)	
History of coronary heart disease (n (%))^b^					< 0.001
No	27987(92.4)	20951(92.8)	1178(89.5)	5858(91.7)	
Yes	2288(7.6)	1619(7.2)	139(10.5)	530(8.3)	

^a^These variables were compared between three groups using *analysis of variance*

^b^These variables were compared between three groups using *chi-square test*

*SD* standard deviation, *CVD* cardiovascular diseases

### The important macronutrients for each type of grains

Additional file 1: Table S4 shows the amount of macronutrient for each type of grains. Wheat and total rice had a similar amount of energy (344 kcal/100 g and 346 kcal/100 g) and carbohydrates (71.5 g/100 g and 71.8 g/100 g). Total rice had a less amount of fat (0.9 g/100 g). Coarse cereals were the main source of dietary fiber with a mean of 5.7 g/100 g.

### Independent and interactional associations of types of grains and lifestyle profiles with all-cause mortality

As adjusting for lifestyle profiles and other covariates, the hazards ratios when adults of total rice and coarse cereals as staple food were compared with adults of wheat as staple food were 0.92 (*95% CI* 0.89, 0.96) and 0.89 (*95% CI* 0.82, 0.96) for all-cause mortality. Compared to participants with healthy lifestyle, those with intermediate lifestyle had a 10% (*HR*: 1.10, *95% CI* 1.06, 1.14) higher all-cause mortality, and those with unhealthy lifestyle had a 14% (*HR*: 1.14, *95% CI* 1.08, 1.20) higher all-cause mortality. Furthermore, similar associations were observed in males and those aged 65–79 years. Meanwhile, multiplicative interactions between types of grains and lifestyle profiles on all-cause mortality were observed in total samples (*P* for interaction = 0.01), females (*P* for interaction = 0.01), and those aged 80 years or older (*P* for interaction = 0.04). However, significant additive interaction was observed only in females (*RERI*: − 0.33, *95% CI* − 0.62, − 0.04), as shown in Table 2.

**Table 2:** Associations of types of grains and lifestyle profiles with all-cause mortality

Subgroups	No of events/participants	*HR* (*95% CI*)	*P*	*P* for interaction ^d^	*RERI* (*95% CI*) ^e^
Total samples (N = 30275)^a^				0.01	− 0.06 (− 0.19,0.07)
Types of grains					
Wheat	4140/6388	1.00			
Total rice	14255/22570	0.92(0.89,0.96)	< 0.001		
Coarse cereals	866/1317	0.89(0.82,0.96)	0.003		
Lifestyle profiles					
Healthy	4768/8385	1.00			
Intermediate	12187/18268	1.10(1.06,1.14)	< 0.001		
Unhealthy	2306/3622	1.14(1.08,1.20)	< 0.001		
Males (N = 13029)^b^				0.09	− 0.06 (− 0.22, 0.10)
Types of grains					
Wheat	1751/2813	1.00			
Total rice	6036/9681	0.89(0.84,0.94)	< 0.001		
Coarse cereals	349/535	0.92(0.82,1.04)	0.17		
Lifestyle profiles					
Healthy	2396/4161	1.00			
Intermediate	3810/5791	1.08(1.02,1.15)	0.01		
Unhealthy	1930/3077	1.13(1.06,1.20)	< 0.001		
Females (N = 17246)^b^				0.01	− 0.33 (− 0.62,− 0.04)
Types of grains					
Wheat	2389/3575	1.00			
Total rice	8219/12889	0.96(0.91,1.01)	0.09		
Coarse cereals	517/782	0.87(0.79,0.97)	0.01		
Lifestyle profiles					
Healthy	2372/4224	1.00			
Intermediate	8377/12477	1.10(1.05,1.16)	< 0.001		
Unhealthy	376/545	1.10(0.97,1.25)	0.14		
Age 65–79 years (N = 6789)^c^				0.63	0.05 (− 0.17, 0.27)
Types of grains					
Wheat	509/1518	1.00			
Total rice	1709/4988	0.83(0.78,0.89)	< 0.001		
Coarse cereals	101/283	0.86(0.75,0.97)	0.02		
Lifestyle profiles					
Healthy	657/2180	1.00			
Intermediate	1250/3500	1.03(0.96,1.09)	0.42		
Unhealthy	412/1109	1.09(1.00,1.18)	0.04		
Age ≥ 80 years (N = 23486)^c^				0.04	− 0.09 (− 0.24, 0.07)
Types of grains					
Wheat	3631/4870	1.00			
Total rice	12546/17582	0.96(0.91,1.01)	0.09		
Coarse cereals	765/1034	0.90(0.82,1.00)	0.04		
Lifestyle profiles					
Healthy	4111/6205	1.00			
Intermediate	10937/14768	1.14(1.09,1.20)	< 0.001		
Unhealthy	1894/2513	1.10(1.02,1.17)	0.01		

^a^Age, sex, body weight, education levels, marital status, living areas, ethnicity, intake of grains, history of hypertension, history of diabetes, history of cardiovascular disease, history of coronary heart disease, and self-reported health were adjusted

^b^Age, body weight, education levels, marital status, living areas, ethnicity, intake of grains, history of hypertension, history of diabetes, history of cardiovascular disease, history of coronary heart disease, and self-reported health were adjusted

^c^Sex, body weight, education levels, marital status, living areas, ethnicity, intake of grains, history of hypertension, history of diabetes, history of cardiovascular disease, history of coronary heart disease, and self-reported health were adjusted

^d^*P* for interaction indicates the multiplicative interaction between types of grains and lifestyle profile on all-cause mortality

^e^RERI was used to evaluate additive interaction between types of grains (total rice vs wheat) and lifestyle profiles (healthy vs unhealthy) on all-cause mortality, and the additive interaction was statistically significant when its confidence interval did not include 0

*RERI* relative excess risk due to interaction

**Fig. 2:**
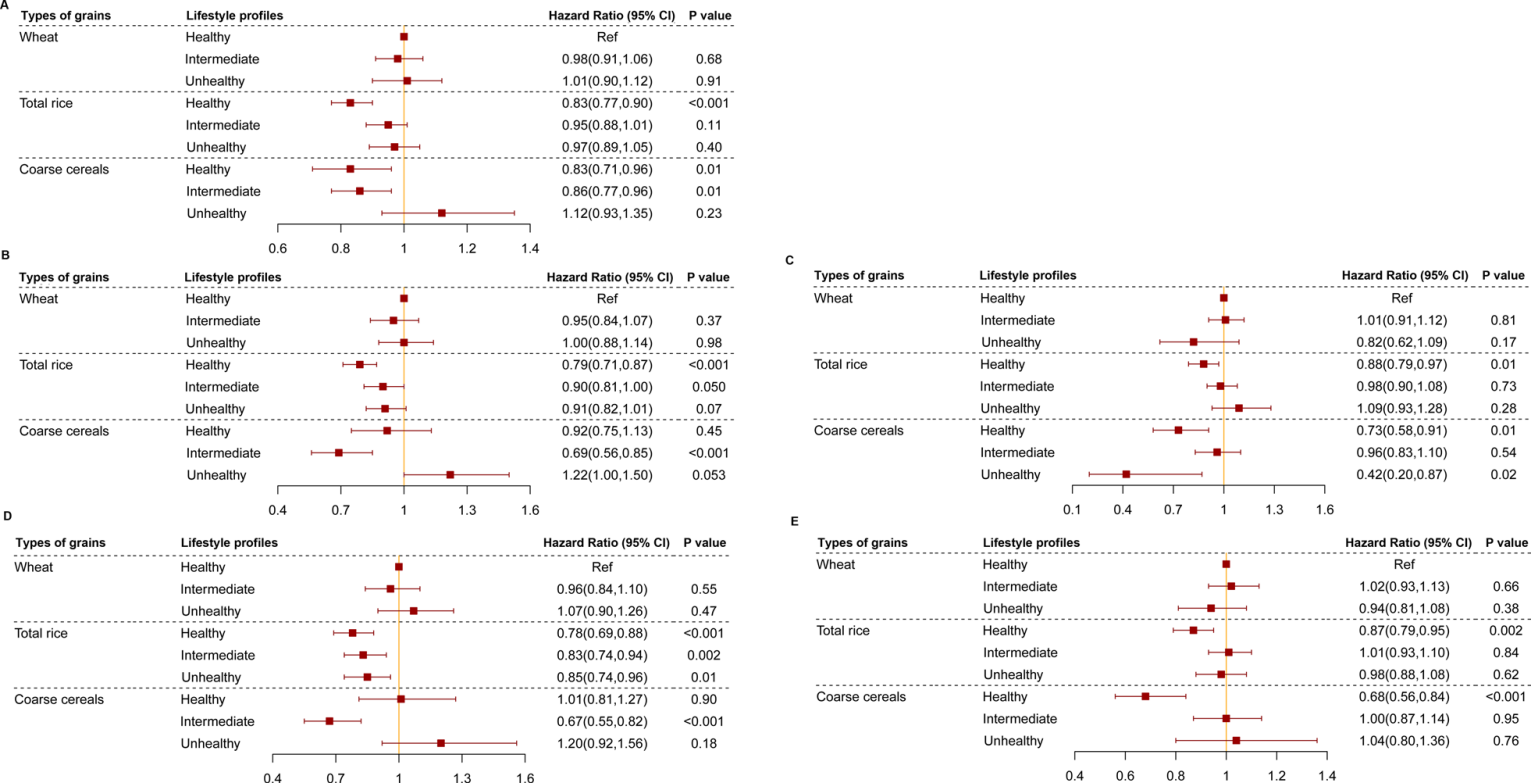
The joint association of types of grains and lifestyle profile with all-cause mortality. **A**: Total samples; **B**: Males; **C**: Females; **D**: Age 65–79 years; and **E**: Age ≥ 80 years

Figure 2 shows the joint associations of types of grains and lifestyle profiles with all-cause mortality. Compared to those with wheat and healthy lifestyle, those with coarse cereals and healthy lifestyle had the lowest risk of all-cause mortality (*HR*: 0.83, *95% CI* 0.71, 0.96), followed by those with total rice and healthy lifestyle (*HR*: 0.83, *95% CI* 0.77, 0.90) (Fig. 2A).

### Associations of intakes of grains with all-cause mortality

Table 3 shows that a 14% higher mortality rate was observed in relation to an increase per SD in intake of coarse cereals (*HR*: 1.14, *95% CI* 1.06, 1.22), whereas null associations were found for intakes of wheat, total rice, and total grains (*P* = 0.76, 0.39, and 0.78). In subgroups, an inverse association of intake of wheat with all-cause mortality was observed in males (*HR*: 0.93, *95% CI* 0.89, 0.97) and those aged 80 years or older (*HR*: 0.95, *95% CI* 0.91, 0.99). A higher mortality rate was observed in relation to a more intake of coarse cereals in females (*HR*: 1.29, *95% CI* 1.17, 1.44) and those aged 80 years or older (*HR*: 1.15, *95% CI* 1.05, 1.26).

**Table 3:** Associations of intakes of grains (per SD) with all-cause mortality

Subgroups	*HR*	*95% CI*	*P*
Total samples (N = 30275)^a^			
Wheat	1.00	0.96,1.03	0.76
Total rice	0.99	0.97,1.01	0.39
Coarse cereals	1.14	1.06,1.22	< 0.001
Total grains	1.00	0.99,1.02	0.78
Males (N = 13029)^b^			
Wheat	0.93	0.89,0.97	0.001
Total rice	1.01	0.98,1.04	0.64
Coarse cereals	0.99	0.89,1.10	0.90
Total grains	0.99	0.96,1.01	0.17
Females (N = 17246)^b^			
Wheat	1.13	1.08,1.19	< 0.001
Total rice	0.98	0.95,1.00	0.08
Coarse cereals	1.29	1.17,1.44	< 0.001
Total grains	1.02	1.00,1.05	0.053
Age 65–79 years (N = 6789)^c^			
Wheat	1.03	0.98,1.08	0.26
Total rice	0.92	0.89,0.95	< 0.001
Coarse cereals	1.11	0.98,1.26	0.10
Total grains	0.96	0.93,0.98	0.001
Age ≥ 80 years (N = 23486)^c^			
Wheat	0.95	0.91,0.99	0.02
Total rice	1.00	0.98,1.03	0.83
Coarse cereals	1.15	1.05,1.26	0.003
Total grains	1.00	0.98,1.02	0.91

^a^Age, sex, body weight, education levels, marital status, living areas, ethnicity, lifestyle profile, history of hypertension, history of diabetes, history of cardiovascular disease, history of coronary heart disease, and self-reported health were adjusted. When total grains as exposure, types of grains was additionally adjusted

^b^Age, body weight, education levels, marital status, living areas, ethnicity, lifestyle profile, history of hypertension, history of diabetes, history of cardiovascular disease, history of coronary heart disease, and self-reported health were adjusted. When total grains as exposure, types of grains was additionally adjusted

^c^Sex, body weight, education levels, marital status, living areas, ethnicity, lifestyle profile, history of hypertension, history of diabetes, history of cardiovascular disease, history of coronary heart disease, and self-reported health were adjusted. When total grains as exposure, types of grains was additionally adjusted

### Associations of types and intakes of grains with all-cause mortality by lifestyle profiles

Compared to wheat as staple food, total rice was associated with a 13% (*HR*: 0.87, *95% CI* 0.80, 0.93) and 6% (*HR*: 0.94, *95% CI* 0.90, 1.00) lower all-cause mortality in participants with healthy and intermediate lifestyle. Meanwhile, a 14% and 12% lower mortality rate was observed in relation to coarse cereals as staple food in those with healthy (*HR*: 0.86, *95% CI* 0.74, 1.00) and intermediate lifestyle (*HR*: 0.88, *95% CI* 0.79, 0.97). On the other hand, an increase per SD in intake of wheat was associated with a 10% (*HR*: 1.10, *95% CI* 1.03, 1.18) higher mortality rate in those with healthy lifestyle. An increase per SD in intake of coarse cereals was linked to a 25% and 17% higher risk of all-cause mortality in those with healthy (*HR*: 1.25, *95% CI* 1.08, 1.44) and intermediate lifestyle (*HR*: 1.17, *95% CI* 1.07, 1.29). However, intake of total rice was related to a decreased all-cause mortality in those with intermediate lifestyle (*HR*: 0.97, *95% CI* 0.94, 0.99), as shown in Table 4.

**Table 4:** Associations of types and intakes (per SD) of grains with all-cause mortality by lifestyle profile (N = 30275)

Exposures	Healthy lifestyle		Intermediate lifestyle		Unhealthy lifestyle	
	*HR* (*95% CI*)	*P*	*HR* (*95% CI*)	*P*	*HR* (*95% CI*)	*P*
Types of grains^a^						
Wheat	1.00		1.00		1.00	
Total rice	0.87(0.80,0.93)	< 0.001	0.94(0.90,1.00)	0.03	0.93(0.84,1.04)	0.20
Coarse cereals	0.86(0.74,1.00)	0.046	0.88(0.79,0.97)	0.01	1.14(0.94,1.39)	0.20
Intakes of grains^b^						
Wheat	1.10(1.03,1.18)	0.01	0.99(0.95,1.04)	0.63	0.94(0.88,1.01)	0.10
Total rice	1.01(0.97,1.05)	0.66	0.97(0.94,0.99)	0.01	1.04(0.99,1.09)	0.09
Coarse cereals	1.25(1.08,1.44)	0.002	1.17(1.07,1.29)	0.001	0.82(0.67,1.00)	0.047
Total grains	1.05(1.01,1.08)	0.01	0.98(0.96,1.00)	0.10	1.00(0.97,1.04)	0.81

^a^Age, sex, body weight, education levels, marital status, living areas, ethnicity, intake of grains, history of hypertension, history of diabetes, history of cardiovascular disease, history of coronary heart disease, and self-reported health were adjusted

^b^Age, sex, body weight, education levels, marital status, living areas, ethnicity, history of hypertension, history of diabetes, history of cardiovascular disease, history of coronary heart disease, and self-reported health were adjusted. When total grains as exposure, types of grains was additionally adjusted

### The nonlinear associations of intakes of grains with all-cause mortality

In Additional file 2: Figure S1, a J-shaped association of intake of wheat with all-cause mortality was observed in females (*P* for non-linearity = 0.002, as shown in Additional file 2: Figure S1A3) and an inversed U-shaped association was observed in those aged 65–79 years (*P* for non-linearity = 0.03, as shown in Additional file 2: Figure S1A4).

In Additional file 2: Figure S1 B1, the hazard ratio of all-cause mortality decreased rapidly until around 350 g/d of total rice intake and then remained relatively flat afterwards, which was considered a J-shaped association (*P* for non-linearity = 0.003). Meanwhile, a J-shaped association of intake of total rice with all-cause mortality was found in females (*P* for non-linearity = 0.03, as shown in Additional file 2: Figure S1B3) and a U-shaped association was observed at an advanced age (*P* for non-linearity < 0.001, as shown in Additional file 2: Figure S1B5). As stratified by lifestyle profiles, a J-shaped association of intake of total rice with all-cause mortality was observed in intermediate lifestyle (*P* for non-linearity = 0.003, as shown in Additional file 2: Figure S1B7).

However, the nonlinear association of intake of coarse cereals with all-cause mortality was not evident, except a J-shaped association of intake of coarse cereals with all-cause mortality in males (*P* for non-linearity = 0.04, as shown in Additional file 2: Figure S1C2).

When combining wheat, total rice, and coarse cereals into total grains, a U-shaped association of intake of total grains with all- cause mortality was observed (*P* for non-linearity = 0.002) in total samples. The hazard ratio of all-cause mortality substantially decreased with an increase in intake of total grains, and reached the lowest risk around intake of 350 g/d, then increased thereafter (Additional file 2: Figure S1D1). As stratified by sex and age, a similar U-shaped association of intake of total grains with all-cause mortality persisted in females (*P* for non-linearity < 0.001, as shown in Additional file 2: Figure S1D3) and at an advanced age (*P* for non-linearity = 0.001, as shown in Additional file 2: Figure S1D5). As stratified by lifestyle profiles, a J-shaped association of intake of total grains with all-cause mortality was observed in intermediate lifestyle (*P* for non-linearity < 0.001, as shown in Additional file 2: Figure S1D7). Additional file 1: Table S5 summaries the finding of the non-linear associations of intakes of grains with all-cause mortality.

### Sensitivity analysis

When excluding participants having chronic diseases, most of results were consistent with the primary outcomes, except the insignificant associations of total rice and coarse cereals in those with intermediate lifestyle and intake of wheat in those with healthy lifestyle with all-cause mortality, as shown in Additional file 1: Table S6. When excluding participants died within 2 year after baseline, the results were comparable with the main analysis, except the significant relationship between total rice and all-cause mortality in those with unhealthy lifestyle as shown in Additional file 1: Table S7. After imputing missing data, the results of complete dataset did not substantially change, as shown in Additional file 1: Table S8.

## Discussion

In this community-based cohort study, compared to wheat, total rice and coarse cereals might be advanced grains and linked to a decreased risk of all-cause mortality. Meanwhile, excessive intake of coarse cereals was related to a higher risk of all-cause mortality. Furthermore, lifestyle profiles modified the relationships between types and intakes of grains and all-cause mortality. Excessive intakes of coarse cereals and wheat were linked to an increased risk of all-cause mortality in those with healthy and intermediate lifestyle. Furthermore, a J-shaped association of intake of total rice with all-cause mortality and a U-shaped association of intake of total grains with all-cause mortality were found.

In China, the consumption of refined grains is increasing [28, 29]. The consumption of wheat has been increasing from 1991 to 2011 [1]. A previous study declared that wheat intake was related to an increased higher risk of CVD [30]. Since the refining process removes a lot of the outer bran layer and pounds the endosperm, intake of refined grains could increase post-prandial blood glucose levels, which are mainly attributed to the repaid action of digestive enzymes and quick absorption of the small intestines [31, 32]. Elevated glucose levels can upregulate the insulin levels, which in turn upregulate hunger and food intake [1]. It was estimated that wheat flours or products per 100 g contain 263 kcal of energy and 49 g of carbohydrate and contribute to 35% energy consumption, whereas white rice per 100 g contains 130 kcal of energy and 28 g of carbohydrate and contributes to 26% energy consumption [1]. Furthermore, the hazard ratios of mortality being attributed to increases per 200 kcal in energy from wheat and white rice were 1.05 (*95% CI* 1.03, 1.07) and 0.98 (*95% CI* 0.96, 1.00), respectively. Furthermore, rice has distinct glycemic and nutritional advantages over wheat products. Therefore, it is interpretable that total rice was linked to a lower mortality rate compared to wheat. In addition, total rice was associated with all-cause mortality only in males and those aged 65–79 years. It is well known that glycemic index of rice is higher than that of wheat, and dietary glycemic index is inversely linked to the risk of all-cause in males but not females, which support the finding of this study [33, 34]. Furthermore, a previous study did not find significant difference between rice and wheat in the association with all-mortality in participants aged 80 years or older, which was consistent with the present study. [35] In addition to total rice, coarse cereals were linked to a decreased all-cause mortality in this study. The potential reasons were that coarse cereals are rich in dietary fiber and vitamins B, which can improve endothelial functions and vascular health [36]. Previous study found that coarse cereals shared nutritional advantages with whole grains and were inversely associated with blood pressure [37].

On the other hand, this study suggested that intakes of total rice and wheat were unrelated to all-cause mortality, which was in line with previous study [1]. However, the conclusion on the relationship between refined grains and all-cause mortality is not well documented. A meta-analysis found no significant association of refined grains with mortality [2]. However, another meta-analysis found a positive association [38]. Meanwhile, a prospective cohort study showed a positive relationship between wheat intake and all-cause mortality, but focused on subjects aged 35 to 70 years, which was different from the current study [1]. Up to date, there were limited studies to assess the impacts of specific types of grains including rice and wheat on mortality. A study in Japan found a protective effect of rice on CVD in males [39]. A cohort study found a positive association of rice and wheat consumption, combined but not separate, with coronary heart disease in Chinese people aged 40–74 years [40]. A recent meta-analysis provided an evidence of an inverse association of rice intake with mortality in males but a positive association in females [41]. However, another meta-analysis found an insignificant relationship between rice intake and CVD mortality [42]. Three US cohorts were used to examine the association of white and brown rice consumption with risk of CVD and found no association, which partly explained the findings of this study [43]. Notably, this study found a positive relationship between coarse cereals consumption and all-cause mortality. Whereas a cross-sectional study found an inverse relationship between coarse grain intake and the risk of hypertension, but it only limited to intake frequency but not intake amount [44]. Furthermore, a U-shaped association of intake of total grains with all-cause mortality and a J-shaped association of intake of total rice with all-casue mortality were found in this study. It suggested that there was a U-shaped relationship between carbohydrate intake and mortality with both low and high carbohydrate consumptions accompanied with an increased mortality risk but intermediate carbohydrate consumption accompanied with a decreased mortality risk [45]. Since the majority of carbohydrates are from processed grains including wheat and rice in China, the relationship between carbohydrate intake and mortality partly expalined the finding of this study [46]. Meanwhile, the lowest risk of all-cause mortality was observed at 350 g/d of total grains and total rice intakes in this study, which also was close to the recommended nutrient intake of grains of the Chinese Dietary Guidelines [47].

In addition, this study found a significant interaction between types of grains and lifestyle profiles on all-cause mortality. A significant association of wheat with all-cause mortality was present in those with healthy and intermediate lifestyle. Meanwhile, intakes of total grains and wheat were linked to a higher risk of all-cause mortality only in those with healthy lifestyle. Therefore, lifestyle profiles could modify the associations of types and intakes of grains with all-cause mortality. It was documented that grain intake was related to lifestyle profiles [48]. Furthermore, low carbohydrate diets were more likely to accompany with lower intakes of vegetables and fruits, which were used to define lifestyle profiles in this study [45]. As a result, wheat, as a relatively high carbohydrate diet, might be consumed more in those with healthy lifestyle than that in those with unhealthy lifestyle. Therefore, it is feasible that intake of wheat affected all-cause mortality only in those with healthy lifestyle. However, the underlying mechanisms on how lifestyle profiles modified the associations of types and intakes of grains with all-cause mortality remained unclear. Further evidences from laboratory are need in the future.

## Strengths and limitations

Advantages of this China-wide survey included the prospective nature, large sample size of older adults, long follow-up period, the combination of 4 lifestyle factors instead of single factors, and the associations of types of grains and the dose–response associations of intakes of grains with all-cause mortality among older adults. However, several limitations also need to be acknowledged. First, since further information on whole grains or refined grains for specific types of grains were unknown, this study failed to adjust fully for the potential confounders. However, rice and wheat products are the major components of grains and often known as refined grains in China [46]. Therefore, the large proportion of refined grains could persist the robustness of the main results. Second, only types of grains and lifestyle profiles at baseline were analyzed in this study. The changes over time could not be captured. However, according to the traditional custom of China, types of grains do not often change, especially in the elderly. Third, since types and intakes of grains were self-reported, there might be recall bias. Fourth, since no data of total energy were collected in the CLHLS, the influence of total energy cannot be adjusted. However, the main sources of total energy have been included and taken into account in the analyses, including grains, vegetables, fruits, eggs, fish, and other diets. Therefore, the residual influence of total energy might be slight.

## Conclusions

Specific types of grains and lifestyle profiles were separately or jointly associated with all-cause mortality. Compared to wheat as staple food, total rice and coarse cereals were linked to a lower risk of all-cause mortality, especially in participants with healthy and intermediate lifestyle. Furthermore, participants with total rice or coarse cereals as staple food and healthy lifestyle had the lowest risk of mortality. Meanwhile, intakes of total grains and total rice were related to all-cause mortality in a dose–response manner. A U-shaped association of intake of total grains with all-cause mortality and a J-shaped association with intake of total rice were found. Therefore, a combination of total rice as major form in grain consumption and intermediate intake with healthy lifestyle should be encouraged among older adults. These findings suggest that future epidemiological research and behavioral interventions should take fully into account the joint associations of types of grains and lifestyle profiles with health outcomes. An understanding of how a broad combination of grains intake and lifestyle factors affects health outcomes could contribute to future public health policy and new intervention targets at individual and population levels.

## Supplementary Information

**Additional file 1**: **Table S1**. Assessment of lifestyle factors. **Table S2** Associations of specific types of diet with all-cause mortality. **Table S3** Baseline characteristics of participants included or excluded from analyses due to missing data. **Table S4** The amount of macronutrient for each type of grains per 100g^a^. **Table S5** The summarized finding of the non-linear associations of intakes of grains with all-cause mortality. **Table S6** Associations of types and intakes of grains and lifestyle profile with all-cause mortality with excluding participants having chronic diseases (N=21021). **Table S7** Associations of types and intakes of grains and lifestyle profile with all-cause mortality with excluding participants died within two years after baseline (N=23239). **Table S8** Associations of types and intakes of grains and lifestyle profile with all-cause mortality using multiple imputation dataset (N=36434).

**Additional file 2**: **Figure S1**. The nonlinear associations of intakes of grains with all-cause mortality. (A1)- (A8) indicate intake of wheat; (B1)- (B8) indicate intake of total rice; (C1)- (C8) indicate intake of coarse cereals; (D1)- (D8) indicate intake of total grains. (A1): Total samples, *P* for non-linearity= 0.72; (A2): Males, *P* for non-linearity= 0.59; (A3): Females, *P* for non-linearity= 0.002; (A4): Age 65-79 years, *P* for non-linearity= 0.03; (A5): Age 80 years or older, *P* for non-linearity= 0.78; (A6): Healthy lifestyle, *P* for non-linearity= 0.66; (A7): Intermediate lifestyle, *P *for non-linearity= 0.43; and (A8): Unhealthy lifestyle, *P* for non-linearity= 0.62. (B1): Total samples, *P* for non-linearity= 0.003; (B2): Males, *P* for non-linearity= 0.10; (B3): Females, *P* for non-linearity= 0.03; (B4): Age 65-79 years, *P* for non-linearity= 0.27; (B5): Age 80 years or older, *P* for non-linearity< 0.001; (B6): Healthy lifestyle, *P* for non-linearity= 0.30; (B7): Intermediate lifestyle, *P *for non-linearity= 0.003; and (B8): Unhealthy lifestyle, *P* for non-linearity= 0.80. (C1): Total samples, *P* for non-linearity= 0.84; (C2): Males, *P* for non-linearity= 0.04; (C3): Females, *P* for non-linearity= 0.83; (C4): Age 65-79 years, *P* for non-linearity= 0.052; (C5): Age 80 years or older, *P* for non-linearity= 0.91; (C6): Healthy lifestyle, *P* for non-linearity= 0.86; (C7): Intermediate lifestyle, *P *for non-linearity= 0.51; and (C8): Unhealthy lifestyle, *P* for non-linearity= 0.94. (D1): Total samples, *P* for non-linearity= 0.002; (D2): Males, *P* for non-linearity= 0.33; (D3): Females, *P* for non-linearity< 0.001; (D4): Age 65-79 years, *P* for non-linearity= 0.10; (D5): Age 80 years or older, *P* for non-linearity= 0.001; (D6): Healthy lifestyle, *P* for non-linearity= 0.07; (D7): Intermediate lifestyle, *P *for non-linearity< 0.001; and (D8): Unhealthy lifestyle, *P* for non-linearity= 0.39.

## Data Availability

The datasets analyzed during the current study are available at the website: https://opendata.pku.edu.cn/dataverse/CHADS.

## Abbreviations

CVD: Cardiovascular disease
CLHLS: Chinese longitudinal healthy longevity survey
BMI: Body mass index
SD: Standard deviation
HRs: Hazard ratios
Cis: Confidence intervals
RERI: Relative excess risk due to interaction
