# Supplementary material for: Associations of types of grains and lifestyle with all-cause mortality among Chinese adults aged 65 years or older: a prospective cohort study
Source: J Transl Med. 2023 Feb 6;21:88. doi: 10.1186/s12967-023-03927-9 (PMC9903469; doi:10.1186/s12967-023-03927-9)
Supplement: Supplementary file 1 — Additional file 1: Table S1. Assessment of lifestyle factors. Table S2 Associations of specific types of diet with all-cause mortality. Table S3 Baseline characteristics of participants included or excluded from analyses due to missing data. Table S4 The amount of macronutrient for each type of grains per 100ga. Table S5 The summarized finding of the non-linear associations of intakes of grains with all-cause mortality. Table S6 Associations of types and intakes of grains and lifestyle profile with all-cause mortality with excluding participants having chronic diseases (N=21021). Table S7 Associations of types and intakes of grains and lifestyle profile with all-cause mortality with excluding participants died within two years after baseline (N=23239). Table S8 Associations of types and intakes of grains and lifestyle profile with all-cause mortality using multiple imputation dataset (N=36434). [file 12967_2023_3927_MOESM1_ESM.docx]

**Supplementary method**

**Assessment of dietary pattern using latent class analysis**

Since fruits, vegetables, fish, eggs, bean products, and tea were associated with all-cause mortality in this study, intake frequencies of these six food were used to generate an overall dietary pattern using latent class analysis. According to the current intake frequencies of these six food, each food group was categorized to “always or almost every day”, “sometimes or occasionally”, and “rarely or never”. Latent class analyses with different numbers of latent classes were conducted to select a reasonable model. The maximum absolute deviation between the parameter estimates in two successive iterations of the estimation procedure was set to 0.000001, and the maximum number of iterations was set to 5000. Akaike information criterion (AIC), Bayesian information criterion (BIC), and likelihood ratio statistic G^2^ were used for the model selection. The mean posterior probability (MPP), which reflects the uncertainty of posterior classification, was also used for the model selection, and a value of 0.75 or more indicates an acceptable uncertainty. Item-response probability is a posterior probability and is used to define latent classes. Table 1 for supplementary method shows G^2^ statistics, AIC, and BIC all continued to go down as more latent classes were added. However, all MPPs from the three-latent-class solution were all≥ 0.80; MPPs of latent classes 1, 2, and 4 from the four-latent-class solution were respectively 0.70, 0.74, and 0.71, which were all less than 0.75; and the MPPs of all latent classes from the five-, six-, and seven-latent-class solution were less than 0.75. Therefore, the three-latent-class solution was the best in terms of the uncertainty of posterior classification. Additionally, as shown in Table 2 for supplementary method, the characteristics of the three-latent-class solution show that latent class 1 was characterized by more intakes of fruits, vegetables, fish, eggs, bean products, and tea, which could be defined as “favorable dietary pattern”; latent class 2 was characterized by fewer intakes of fruits, vegetables, fish, eggs, bean products, and tea, which could be defined as “unfavorable dietary pattern”; latent class 3 was characterized by medium intakes of fruits, vegetables, fish, eggs, bean products, and tea, which could be defined as “intermediate dietary pattern”. Therefore, dietary pattern was categorized as unfavorable, intermediate, or favorable dietary pattern.

Table 1 for supplementary method G^2^ statistics, AIC, BIC, and mean posterior probabilities (MPPs) in models with different numbers of latent classes for dietary pattern

| Number of  latent classes | G-squared | AIC | BIC | MPPs | | | | | | |
| --- | --- | --- | --- | --- | --- | --- | --- | --- | --- | --- |
|  |  |  |  | Latent class 1 | Latent class 2 | Latent class 3 | Latent class 4 | Latent class 5 | Latent class 6 | Latent  class 7 |
| 2 | 10000 | 10000 | 10000 | 0.85 | 0.90 | - | - | - | - | - |
| 3 | 3700 | 3700 | 4100 | 0.82 | 0.80 | 0.82 | - | - | - | - |
| 4 | 3200 | 3300 | 3800 | 0.70 | 0.74 | 0.81 | 0.71 | - | - | - |
| 5 | 2600 | 2700 | 3200 | 0.77 | 0.71 | 0.74 | 0.71 | 0.66 | - | - |
| 6 | 2300 | 2400 | 3100 | 0.73 | 0.67 | 0.69 | 0.63 | 0.50 | 0.72 | - |
| 7 | 2100 | 2200 | 3000 | 0.53 | 0.66 | 0.70 | 0.45 | 0.61 | 0.71 | 0.69 |

Table 2 for supplementary method Prevalence of latent classes and item-response probabilities in models with three latent classes for dietary pattern

| Item ^a^ | Latent class 1 ^b^ | Latent class 2 ^b^ | Latent class 3 ^b^ |
| --- | --- | --- | --- |
| Prevalence | 0.33 | 0.25 | 0.42 |
| Fruits 1 | **0.55** | 0.10 | 0.21 |
| Fruits 2 | 0.34 | 0.39 | **0.61** |
| Fruits 3 | 0.11 | **0.51** | 0.18 |
| Vegetables 1 | **0.96** | 0.79 | 0.86 |
| Vegetables 2 | 0.03 | **0.15** | **0.13** |
| Vegetables 3 | 0.01 | **0.06** | 0.01 |
| Fish 1 | **0.43** | 0.08 | 0.05 |
| Fish 2 | 0.43 | 0.24 | **0.81** |
| Fish 3 | 0.14 | **0.68** | 0.14 |
| Eggs 1 | **0.76** | 0.17 | 0.14 |
| Eggs 2 | 0.17 | 0.32 | **0.82** |
| Eggs 3 | 0.07 | **0.52** | 0.04 |
| Bean products 1 | **0.66** | 0.18 | 0.11 |
| Bean products 2 | 0.27 | 0.41 | **0.83** |
| Bean products 3 | 0.07 | **0.41** | 0.06 |
| Tea 1 | **0.45** | 0.25 | 0.29 |
| Tea 2 | 0.16 | 0.11 | **0.25** |
| Tea 3 | 0.39 | **0.64** | 0.46 |

^a^ Prevalence indicated the prevalence of each latent class. The first category of each food indicates intake frequency of “always or almost every day”. The second category of each food indicates intake frequency of “sometimes or occasionally”. The third category of each food indicates intake frequency of “rarely or never”.

^b^ The maximal item-response probabilities for each latent class were marked in bold.

**Assessment of lifestyle profiles using latent class analysis**

The lifestyle profile was constructed using smoking, alcohol consumption, physical activity, and dietary pattern, which were categorized to three categories, except alcohol consumption for two categories. According to the criterion mentioned in the assessment of dietary pattern, G^2^ statistics, AIC, BIC, and MPP were used for the model selection. Since the model with five latent classes failed to converge, only information on models with five or fewer latent classes were reported. Table 3 for supplementary method shows G^2^ statistics, AIC, and BIC all continued to go down as more latent classes were added. However, all MPPs from the three-latent-class solution were all≥ 0.75; MPPs of latent classes 2 and 3 from the four-latent-class solution were respectively 0.66 and 0.62, which were less than 0.75. Therefore, the three-latent-class solution was the best in terms of the uncertainty of posterior classification. Additionally, as shown in Table 4 for supplementary method, the characteristics of the three-latent-class solution show that latent class 1 was characterized by current or former smokers, binge/ moderate drinking, no physical activity, and intermediate dietary pattern, which could be defined as “unhealthy lifestyle”; latent class 2 was characterized by never smokers, never drinking, no physical activity, and intermediate or unfavorable dietary pattern, which could be defined as “intermediate lifestyle”; and latent class 3 was characterized by never smokers, never drinking, current or former physical activity, and favorable dietary pattern, which could be defined as “healthy lifestyle”. Therefore, lifestyle profile was categorized as healthy, intermediate, or unhealthy lifestyle.

Table 3 for supplementary method G^2^ statistics, AIC, BIC, and mean posterior probabilities (MPP) in models with different numbers of latent classes for lifestyle profiles

| Number of  latent classes | G-squared | AIC | BIC | MPP | | | | |
| --- | --- | --- | --- | --- | --- | --- | --- | --- |
|  |  |  |  | Latent  class 1 | Latent  class 2 | Latent  class 3 | Latent  class 4 | Latent  class 5 |
| 2 | 1800 | 1800 | 1900 | 0.80 | 0.89 | - | - | - |
| 3 | 400 | 450 | 640 | 0.77 | 0.97 | 0.88 | - | - |
| 4 | 180 | 240 | 500 | 0.81 | 0.66 | 0.62 | 0.79 | - |
| 5  (not converge) | 57 | 130 | 460 | 0.67 | 0.61 | 0.70 | 0.80 | 0.63 |

Table 4 for supplementary method Prevalence of latent classes and item-response probabilities in models with three latent classes for lifestyle profiles

| Item ^a^ | Latent class 1 ^b^ | Latent class 2 ^b^ | Latent class 3 ^b^ |
| --- | --- | --- | --- |
| Prevalence | 0.12 | 0.58 | 0.30 |
| Smoking 1 | **0.79** | 0.17 | 0.18 |
| Smoking 2 | **0.21** | 0.10 | 0.18 |
| Smoking 3 | 0.01 | **0.74** | **0.64** |
| Alcohol consumption 1 | **0.99** | 0.11 | 0.13 |
| Alcohol consumption 2 | 0.01 | **0.89** | **0.87** |
| Physical activity 1 | 0.40 | 0.05 | **0.91** |
| Physical activity 2 | 0.02 | 0.03 | **0.07** |
| Physical activity 3 | **0.58** | **0.93** | 0.02 |
| Dietary pattern 1 | 0.36 | 0.25 | **0.48** |
| Dietary pattern 2 | 0.20 | **0.27** | 0.18 |
| Dietary pattern 3 | **0.43** | **0.48** | 0.34 |

^a^ Prevalence indicated the prevalence of each latent class. Smoking 1 to 3 respectively referred to current, former, or never smokers. Alcohol consumption 1 to 2 respectively referred to binge/ moderate or never drinking. Physical activity 1 to 3 respectively referred to current, former, or never physical activity. Dietary pattern 1 to 3 respectively referred to as favorable, unfavorable, or intermediate dietary pattern.

^b^ The maximal item-response probabilities for each latent class were marked in bold.

Supplementary Table S1 Assessment of lifestyle factors

| Lifestyle factors | Assessment methods | Lifestyle status |
| --- | --- | --- |
| Smoking | Question 1: Smoke or not at present? | 1. Current smokers: Yes for Q1 regardless of Q2.  2. Former smokers: No for Q1 and Yes for Q2.  3. Never smokers: No for both Q1 and Q2. |
|  | Question 2: Smoked or not in the past? |  |
| Alcohol consumption | Question 1: Drink or not at present? | 1. Binge drinkers: men drank> 25 g/d of alcohol or women drank> 15 g/d of alcohol at present or in the past.  2. Moderate drinkers: men drank≤ 25 g/d of alcohol or women drank≤ 15 g/d of alcohol at present or in the past.  3. Never drinkers: No for both Q1 and Q2. |
|  | Question 2: Drank or not in the past? |  |
|  | Question 3: How much do you drink on average every day if drink or drank? |  |
| Physical activity | Question 1: Exercise or not at present? | 1. Current physical activity: Yes for Q1 regardless of Q2.  2. Former physical activity: No for Q1 and Yes for Q2.  3. Never physical activity: No for both Q1 and Q2. |
|  | Question 2: Exercised or not in the past? |  |

Supplementary Table S2 Associations of specific types of diet with all-cause mortality

| Specific types of diet ^a^ | *HR* | *95% CI* | *P* |
| --- | --- | --- | --- |
| **Fruits** |  |  |  |
| Always or almost every day | 1.00 |  |  |
| Sometimes or occasionally | 1.06 | 1.01,1.10 | 0.01 |
| Rarely or never | 1.12 | 1.07,1.18 | <0.001 |
| **Vegetables** |  |  |  |
| Always or almost every day | 1.00 |  |  |
| Sometimes or occasionally | 0.94 | 0.90,0.99 | 0.01 |
| Rarely or never | 1.15 | 1.04,1.28 | 0.01 |
| **Meat** |  |  |  |
| Always or almost every day | 1.00 |  |  |
| Sometimes or occasionally | 1.03 | 0.99,1.07 | 0.11 |
| Rarely or never | 0.97 | 0.93,1.02 | 0.25 |
| **Fish** |  |  |  |
| Always or almost every day | 1.00 |  |  |
| Sometimes or occasionally | 1.10 | 1.05,1.15 | <0.001 |
| Rarely or never | 1.10 | 1.05,1.16 | <0.001 |
| **Eggs** |  |  |  |
| Always or almost every day | 1.00 |  |  |
| Sometimes or occasionally | 1.10 | 1.06,1.14 | <0.001 |
| Rarely or never | 1.07 | 1.03,1.13 | 0.003 |
| **Bean products** |  |  |  |
| Always or almost every day | 1.00 |  |  |
| Sometimes or occasionally | 1.04 | 1.00,1.08 | 0.053 |
| Rarely or never | 1.04 | 0.99,1.10 | 0.09 |
| **Tea** |  |  |  |
| Always or almost every day | 1.00 |  |  |
| Sometimes or occasionally | 1.06 | 1.01,1.11 | 0.02 |
| Rarely or never | 1.04 | 1.01,1.08 | 0.03 |
| **Salt-preserved vegetables** |  |  |  |
| Always or almost every day | 1.00 |  |  |
| Sometimes or occasionally | 1.01 | 0.97,1.05 | 0.73 |
| Rarely or never | 1.00 | 0.96,1.04 | 0.91 |
| **Sugar** |  |  |  |
| Always or almost every day | 1.00 |  |  |
| Sometimes or occasionally | 0.98 | 0.94,1.02 | 0.31 |
| Rarely or never | 0.97 | 0.93,1.02 | 0.21 |
| **Garlic** |  |  |  |
| Always or almost every day | 1.00 |  |  |
| Sometimes or occasionally | 1.03 | 0.98,1.07 | 0.25 |
| Rarely or never | 1.00 | 0.96,1.05 | 0.96 |

^a^ For each type of diet, age, sex, body weight, education levels, marital status, living areas, ethnicity, types of grains, history of hypertension, history of diabetes, history of cardiovascular disease, history of coronary heart disease, and self-reported health were adjusted.

Supplementary Table S3 Baseline characteristics of participants included or excluded from analyses due to missing data

| Characteristics | Included participants  (N= 30275) | Excluded participants  (N= 5738) |
| --- | --- | --- |
| Age (years, mean ± SD) | 87±11 | 94±10 |
| Body weight (kg, mean ± SD) | 49±11 | 46±11 |
| Intakes of grains  (g/d, mean ± SD) | 300±130 | 280±130 |
| Sex (n (%)) |  |  |
| Male | 13029(43) | 1833(32) |
| Female | 17246(57) | 3905(68) |
| Marital status (n (%)) |  |  |
| Married | 9158(30) | 941(16) |
| Divorced | 153(0.5) | 21(0.4) |
| Widowed | 20632(68) | 4707(82) |
| Unmarried | 332(1.1) | 62(1.1) |
| Living areas (n (%)) |  |  |
| Urban | 12173(40) | 2155(38) |
| Rural | 18102(60) | 3583(62) |
| Education (n (%)) |  |  |
| Illiteracy | 19138(63) | 4152(73) |
| Primary school or above | 11137(37) | 1567(27) |
| Ethnicity (n (%)) |  |  |
| Han | 28283(93.4) | 5362(93.5) |
| Others | 1992(6.6) | 376(6.5) |
| Smoking status (n (%)) |  |  |
| Current smoker | 5747(19) | 830(15) |
| Former smoker | 4240(14) | 699(12) |
| Never smoker | 20288(67) | 4176(73) |
| Alcohol consumer status  (n (%)) |  |  |
| Yes | 6280(21) | 941(17) |
| No | 23995(79) | 4584(83) |
| Physical activity (n (%)) |  |  |
| Current | 8765(29) | 1044(18) |
| Former | 1993(6.6) | 515(9.1) |
| Never | 19517(64) | 4109(72) |
| Dietary pattern (n (%)) |  |  |
| Unfavorable | 7426(25) | 1347(26) |
| Intermediate | 13506(45) | 1591(31) |
| Favorable | 9343(30) | 2264(43) |
| Lifestyle profiles (n (%)) |  |  |
| Unhealthy | 3622(12) | 1013(19) |
| Intermediate | 18268(60) | 3774(73) |
| Healthy | 8385(28) | 415(8) |
| Self-reported health (n (%)) |  |  |
| Poor | 3857(13) | 371(13) |
| Fair | 10230(34) | 1011(37) |
| Good | 16188(53) | 1361(50) |
| History of hypertension  (n (%)) |  |  |
| No | 12665(42) | 2346(41) |
| Yes | 17610(58) | 3392(59) |
| History of diabetes (n (%)) |  |  |
| No | 29721(98.2) | 3932(98.3) |
| Yes | 554(1.8) | 70(1.7) |
| History of CVD (n (%)) |  |  |
| No | 29106(96.1) | 3859(92.8) |
| Yes | 1169(3.9) | 299(7.2) |
| History of coronary  heart disease (n (%)) |  |  |
| No | 27987(92.4) | 3730(90.8) |
| Yes | 2288(7.6) | 378(9.2) |

SD: standard deviation, CVD: cardiovascular diseases

Supplementary Table S4 The amount of macronutrient for each type of grains per 100g ^a^

| Grains | Energy (kcal) | Water (g) | Protein (g) | Fat (g) | Dietary fiber (g) | Carbohydrate (g) |
| --- | --- | --- | --- | --- | --- | --- |
| **Wheat** | 344 | 12.7 | 11.2 | 1.5 | 2.1 | 71.5 |
| Wheat flour | 392 | 4.3 | 36.4 | 10.1 | 5.6 | 38.9 |
| Steamed bread | 233 | 40.5 | 7.8 | 1.0 | 1.5 | 48.3 |
| Noodles | 355 | 10.5 | 11.0 | 0.1 | 0.2 | 77.5 |
| **Total rice** | 346 | 12.9 | 12.7 | 0.9 | 0.6 | 71.8 |
| Cooked rice | 117 | 70.6 | 2.6 | 0.3 | 0.2 | 26.0 |
| Rice noodles | 346 | 12.3 | 8.0 | 0.1 | 0.1 | 78.2 |
| **Coarse cereals (mean)** | 335 | 12.2 | 9.5 | 2.7 | 5.7 | 68.0 |
| Maize | 335 | 13.2 | 8.7 | 3.8 | 6.4 | 66.6 |
| Millet | 358 | 11.6 | 9.0 | 3.1 | 1.6 | 73.5 |
| Buckwheat | 324 | 13.0 | 9.3 | 2.3 | 6.5 | 66.5 |
| Sorghum | 351 | 10.3 | 10.4 | 3.1 | 4.3 | 70.4 |
| Barley | 307 | 13.1 | 10.2 | 1.4 | 9.9 | 63.4 |

^a^ Values taken from the Chinese food ingredient list 2002 (https://www.cnsoc.org/).

Supplementary Table S5 The summarized finding of the non-linear associations of intakes of grains with all-cause mortality

| Subgroups | Wheat | | | Total rice | | | Coarse cereals | | | Total grains | | |
| --- | --- | --- | --- | --- | --- | --- | --- | --- | --- | --- | --- | --- |
|  | Nonlinear | RNI (g/d) | Shapes | Nonlinear | RNI (g/d) | Shapes | Nonlinear | RNI (g/d) | Shapes | Nonlinear | RNI (g/d) | Shapes |
| Total samples | No | N.A | Linear | Yes | 250-350 | “J” | No | N.A | Linear | Yes | 250-350 | “U” |
| Males | No | N.A | Linear | No | N.A | Linear | Yes | 250-400 | “J” | No | N.A | Linear |
| Females | Yes | <350 | “J” | Yes | 250-320 | “J” | No | N.A | Linear | Yes | 250-300 | “U” |
| Age 65-79 years | Yes | <250 | Inversed “U” | No | N.A | Linear | No | N.A | Linear | No | N.A | Linear |
| Age≥ 80 years | No | N.A | Linear | Yes | 250-300 | “U” | No | N.A | Linear | Yes | 250-300 | “U” |
| Healthy lifestyle | No | N.A | Linear | No | N.A | Linear | No | N.A | Linear | No | N.A | Linear |
| Intermediate lifestyle | No | N.A | Linear | Yes | 250-400 | “J” | No | N.A | Linear | Yes | 250-400 | “J” |
| Unhealthy lifestyle | No | N.A | Linear | No | N.A | Linear | No | N.A | Linear | No | N.A | Linear |

RNI: recommended nutrient intake.

Supplementary Table S6 Associations of types and intakes of grains and lifestyle profile with all-cause mortality with excluding participants having chronic diseases (N=21021)

| Subgroups | Total samples | | Healthy lifestyle | | Intermediate lifestyle | | Unhealthy lifestyle | |
| --- | --- | --- | --- | --- | --- | --- | --- | --- |
|  | *HR* (*95% CI*) | *P* | *HR* (*95% CI*) | *P* | *HR* (*95% CI*) | *P* | *HR* (*95% CI*) | *P* |
| **Types of grains ^a^** |  |  |  |  |  |  |  |  |
| Wheat | 1.00 |  | 1.00 |  | 1.00 |  | 1.00 |  |
| Total rice | 0.94(0.90,0.99) | 0.01 | 0.85(0.77,0.94) | 0.001 | 0.96(0.90,1.03) | 0.27 | 0.97(0.86,1.10) | 0.65 |
| Coarse cereals | 0.89(0.81,0.97) | 0.01 | 0.83(0.69,1.00) | 0.053 | 0.91(0.80,1.03) | 0.13 | 1.05(0.83,1.33) | 0.69 |
| **Intakes of grains ^b^** |  |  |  |  |  |  |  |  |
| Wheat | 0.99(0.95,1.03) | 0.72 | 1.06(0.97,1.15) | 0.21 | 1.01(0.95,1.07) | 0.74 | 0.93(0.86,1.01) | 0.09 |
| Total rice | 0.98(0.96,1.01) | 0.20 | 0.98(0.93,1.03) | 0.49 | 0.96(0.93,1.00) | 0.02 | 1.03(0.97,1.09) | 0.31 |
| Coarse cereals | 1.14(1.05,1.25) | 0.002 | 1.25(1.03,1.51) | 0.02 | 1.20(1.07,1.35) | 0.002 | 0.76(0.60,0.95) | 0.02 |
| Total grains | 1.00(0.98,1.02) | 0.98 | 1.03(0.98,1.07) | 0.23 | 0.99(0.96,1.01) | 0.31 | 1.00(0.96,1.05) | 0.94 |

^a^ Age, sex, body weight, education levels, marital status, living areas, ethnicity, intake of grains, and self-reported health were adjusted. In total sample, lifestyle profile was additionally adjusted.

^b^ Age, sex, body weight, education levels, marital status, living areas, ethnicity, and self-reported health were adjusted. In total sample, lifestyle profile was additionally adjusted.

Supplementary Table S7 Associations of types and intakes of grains and lifestyle profile with all-cause mortality with excluding participants died within two years after baseline (N=23239) ^a^

| Subgroups | Total samples | | Healthy lifestyle | | Intermediate lifestyle | | Unhealthy lifestyle | |
| --- | --- | --- | --- | --- | --- | --- | --- | --- |
|  | *HR* (*95% CI*) | *P* | *HR* (*95% CI*) | *P* | *HR* (*95% CI*) | *P* | *HR* (*95% CI*) | *P* |
| **Types of grains ^a^** |  |  |  |  |  |  |  |  |
| Wheat | 1.00 |  | 1.00 |  | 1.00 |  | 1.00 |  |
| Total rice | 0.91(0.87,0.95) | <0.001 | 0.85(0.78,0.92) | <0.001 | 0.94(0.88,0.99) | 0.03 | 0.88(0.78,0.99) | 0.03 |
| Coarse cereals | 0.87(0.79,0.95) | 0.001 | 0.83(0.70,0.98) | 0.03 | 0.85(0.76,0.96) | 0.01 | 1.15(0.93,1.42) | 0.21 |
| **Intakes of grains ^b^** |  |  |  |  |  |  |  |  |
| Wheat | 1.01(0.97,1.04) | 0.69 | 1.10(1.02,1.19) | 0.01 | 1.01(0.96,1.06) | 0.64 | 0.96(0.89,1.03) | 0.26 |
| Total rice | 0.99(0.97,1.01) | 0.37 | 1.00(0.96,1.05) | 0.90 | 0.96(0.94,0.99) | 0.02 | 1.04(0.99,1.10) | 0.12 |
| Coarse cereals | 1.13(1.04,1.22) | 0.003 | 1.08(0.91,1.28) | 0.38 | 1.23(1.10,1.38) | <0.001 | 0.84(0.67,1.05) | 0.13 |
| Total grains | 1.01(0.99,1.02) | 0.58 | 1.04(1.00,1.07) | 0.07 | 0.99(0.96,1.01) | 0.35 | 1.01(0.97,1.05) | 0.76 |

^a^ Age, sex, body weight, education levels, marital status, living areas, ethnicity, intake of grains, and self-reported health were adjusted. In total sample, lifestyle profile was additionally adjusted.

^b^ Age, sex, body weight, education levels, marital status, living areas, ethnicity, and self-reported health were adjusted. In total sample, lifestyle profile was additionally adjusted.

Supplementary Table S8 Associations of types and intakes of grains and lifestyle profile with all-cause mortality using multiple imputation dataset (N=36434) ^a^

| Subgroups | Total samples | | Healthy lifestyle | | Intermediate lifestyle | | Unhealthy lifestyle | |
| --- | --- | --- | --- | --- | --- | --- | --- | --- |
|  | *HR* (*95% CI*) | *P* | *HR* (*95% CI*) | *P* | *HR* (*95% CI*) | *P* | *HR* (*95% CI*) | *P* |
| **Types of grains ^a^** |  |  |  |  |  |  |  |  |
| Wheat | 1.00 |  | 1.00 |  | 1.00 |  | 1.00 |  |
| Total rice | 0.92(0.89,0.96) | <0.001 | 0.86 (0.80,0.92) | <0.001 | 0.95(0.90,1.00) | 0.047 | 0.93(0.84,1.04) | 0.21 |
| Coarse cereals | 0.89(0.82,0.96) | 0.002 | 0.86(0.74,1.00) | 0.054 | 0.87(0.79,0.97) | 0.01 | 1.12(0.92,1.37) | 0.24 |
| **Intakes of grains ^b^** |  |  |  |  |  |  |  |  |
| Wheat | 0.99(0.96,1.02) | 0.63 | 1.10(1.03,1.18) | 0.01 | 0.99(0.95,1.03) | 0.57 | 0.93(0.87,1.00) | 0.06 |
| Total rice | 0.99(0.97,1.01) | 0.14 | 1.00(0.96,1.04) | 0.93 | 0.97(0.94,0.99) | 0.01 | 1.03(0.98,1.08) | 0.21 |
| Coarse cereals | 1.14(1.06,1.22) | <0.001 | 1.19(1.03,1.36) | 0.02 | 1.16(1.06,1.28) | 0.002 | 0.93(0.77,1.12) | 0.44 |
| Total grains | 1.00(0.98,1.01) | 0.76 | 1.04 (1.01,1.07) | 0.03 | 0.98(0.96,1.00) | 0.09 | 1.00(0.96,1.03) | 0.81 |

^a^ Age, sex, body weight, education levels, marital status, living areas, ethnicity, intake of grains, and self-reported health were adjusted. In total sample, lifestyle profile was additionally adjusted.

^b^ Age, sex, body weight, education levels, marital status, living areas, ethnicity, and self-reported health were adjusted. In total sample, lifestyle profile was additionally adjusted.
